# Disrespect and abuse during childbirth in district Gujrat, Pakistan: A quest for respectful maternity care

**DOI:** 10.1371/journal.pone.0200318

**Published:** 2018-07-11

**Authors:** Zainab Azhar, Oyinlola Oyebode, Haleema Masud

**Affiliations:** 1 Al-Shifa School of Public Health, Al-Shifa Trust Eye Hospital, Rawalpindi, Pakistan; 2 Warwick Medical School, University of Warwick, Coventry, United Kingdom; James Cook Unviersity, AUSTRALIA

## Abstract

**Background:**

Disrespectful and abusive practices at health facilities during childbirth discourage many women to seek care at facilities. This may lead to maternal morbidity and mortalities. Despite severe impacts, such practices remain hidden and are rarely reported in developing countries.

**Objectives:**

The study was carried out to assess the prevalence and determinants of the disrespect and abuse (D & A) during child birth in rural Gujrat, Pakistan.

**Methods:**

A cross sectional household based study was conducted in tehsil Kharian of district Gujrat. Data was collected using an interview based questionnaire from the women who had a live birth within the previous two months (n = 360). The D & A scale was based on standard Maternal and Child Health Integrated Programme indicators. Multiple logistic regression was used to find out the determinants of reported D & A.

**Results:**

Almost all women experienced D & A (99.7%) during childbirth according to objective assessment “experienced D & A”. However, only 27.2% reported subjective experience of D & A “reported D & A”. The main determinant of reported D & A was facility based childbirth (OR = 13.49; 10.10–100.16) and lower socio economic strata (OR = 2.89; 1.63–5.11). The risk of reporting D & A was twice in public health facilities as compared to private. Women who had reported D & A were more likely to opt for changing the place of childbirth for next time (OR = 4.37, 95% CI = 2.41–7.90).

**Conclusion:**

D & A during childbirth is highly prevalent and under-recognized in Pakistan. High prevalence at facilities and particularly at public facilities can be a reason for underutilization of this sector for childbirth. Maternal health policies in Pakistan need to be revised based on the charter of respectful maternity care.

## Introduction

Pakistan is one of the countries with the highest burden of maternal, newborn and infant mortalities in South Asia [1]. Despite formal commitment and implementation of various projects, Pakistan did not achieve any of its health related Millennium Development Goals (MDGs) [2]. Maternal mortality remains high in the country with 260 deaths per 100000 live births, a large way behind its MDG target of 140 [2]. Neonatal mortality is also alarming, Pakistan ranking at number two in Asia with highest neonatal mortality [3]. These deplorable statistics are well explained as only 52% of the births are attended by skilled birth attendants (SBA) and a small proportion (37%) of mothers receive essential antenatal care (ANC) in the country [4]. However, these poor health indicators cannot be linked to lack of health facilities or skilled professionals. In fact, Pakistan enjoys quite extensive network of public and private health facilities [5]. But the utilization of health facilities particularly public facilities for maternity care is quite low. Public sector caters for only 15% of the deliveries in country^4^. Instead people opt for either specialized private sector, which is quite expensive, or relatively cheaper private maternity care providers (midwives or untrained staff). In the worst scenario i.e., in almost 50% of the cases, women opt to stay at home either assisted by a traditional birth attendant (TBA) or a family member while giving birth [4].

Underutilization of facilities cannot be solely attributed to financial and cultural barriers in Pakistan, as almost 61% of the women tend to utilize facilities for the delivery of their first child but not for the subsequent childbirths (51%) [6]. This remarkable difference in utilization for the subsequent deliveries can be partly ascribed to past adverse experiences at health facilities. Facility based maltreatment is reported to be a strong factor dissuading many women from utilizing the available and accessible services [7,8]. Women experience ill treatment not only in violation of their autonomy and dignity but also as verbal insults, humiliation, discrimination, abandonment of care and physical assault during childbirth. Bowser and Hill (2010) formally called these maltreatments Disrespect and Abuse (D&A) during childbirth and highlighted this as a main factor in the underutilization of health care facilities [9]. A series of studies in Africa show a huge prevalence of the problem. A study in a teaching hospital in Nigeria found that almost all (98%) women experienced D & A during childbirth [10]. Statistics from Kenya show that 22.2% of women do not access services due to inappropriate behavior of providers [11]. Evidence suggests that 60% of women in Tanzania would improve their utilization of health facilities if providers show respectful attitudes [12]. Considering the impact of D & A on service utilization, it is recognized as one of the four deterrents to the skilled birth utilization [9].

Although an objective assessor reviewing statements about a woman’s experience during labor and birth may see that she has been a victim of D & A “experienced D & A”, the woman herself may not recognize that this was D & A “reported D & A”. A questionnaire study of 173 mothers in Addis Ababa, Ethiopia, immediately prior to their discharge after childbirth, found that although 78% of women answered “yes” to statements describing D&A during their birth experience (such as “the provider did not explain to me what is being done and what to expect throughout labor and birth” or “the provider did not allow me to move during labor”) just 16.2% reported that they had *felt* disrespected or abused [13]. D & A during child birth is very common but mostly remains under the cover of unawareness and normalization in developing countries [14]. Study and quantification of D & A is warranted to get a deeper understanding of poor maternal health and low services utilization for childbirth in Pakistan. This study was carried out to estimate the prevalence and determinants of D & A during childbirth and also to assess its impact on future decisions about selection of the place of birth.

## Material and methods

A cross sectional, community based study was carried out to assess the level of D & A during childbirth in District Gujrat of Pakistan from August 2015 to March 2016. It is a populous district with over 2.6 million residents [15], 22% of them are women of reproductive age [16]. Health services are provided by both public and private sector in the region. The public sector is comprised of one district hospital, one tehsil hospital, nine Rural Health Centres (RHC), eighty nine Basic Health Units (BHUs) and nine Maternal and Child Health (MCH) centers. In addition, 53 community midwives (CMWs) and 1716 lady health workers (LHWs) are allotted throughout the district to provide home based reproductive health services and primary care [15]. The private sector services range from highly skilled doctors working at high quality hospitals to clinics, to “quacks” (an unqualified person who falsely claims to have medical skills) and TBAs. People from this district also go to the neighboring district, Jhelum to get maternity and other healthcare services.

### Sample size and sampling strategy

A sample size of 359 was calculated by using proportion formula on OpenEpi software version 3 with an expected proportion of D & A, 16.2% at 5% precision and 99% confidence level [13]. The number of annual expected pregnancies (90624) of the district was taken as population [16].

We expected to get the desired sample size from seven Union Councils (UCs). These UCs were randomly selected from *tehsil* Kharian of the district using random number table. All women who have had a live child birth in these UCs during last two months were selected to participate in the study, this made a sample of 360 women. Women who had delivered under general anesthesia and those whose child had died were not included in the study. Lists of these women were obtained from the LHW of every village in the selected Union Councils (UCs).

### Data collection tool

A structured questionnaire, which was bilingual type in both English and Urdu languages, was used for data collection. The data collection tool was based on standard Maternal and Child Health Integrated Programe (MCHIP) indicators developed by USAID and also on findings of studies conducted in Tanzania [12] and Ethiopia [13]. D & A was measured on a 25 itemed scale, comprising of seven categories of D & A: physical abuse, non-consented care, non-confidential care, non-dignified care, discriminatory care, abandonment in facility and detention in facility (Table 1).

**Table 1:** Categories of disrespect and abuse during childbirth.

S.No.	Categories and items of D & A scale
**1**	**Non-consented care**
1.1 1.2 1.3 1.4 1.5 1.6 1.7 1.8	Provider did not introduce herself Provider did not encourage to ask questions Provider didn’t responded politely, truthfully and promptly Provider didn’t explained procedure and explained expectations Provider didn’t give the periodic updates on status and progress Provider didn’t allow to move during delivery Provider didn’t allow to assume position of choice Provider didn’t obtain consent prior to procedure
**2**	**Non confidential care**
2.1 2.2 2.3	Curtains and physical barriers were not used Drape or body covering was not used The number of staff members around were not logical
**3**	**Non-dignified care**
3.1 3.2 3.3	Provider didn’t speak politely Provider made insults, threats etc. Provider used abusive language
**4**	**Discriminatory care**
4.1. 4.2	Provider used language difficult to understand Provider showed disrespect based on specific attribute
**5**	**Abandonment in facility**
5.1 5.2 5.3	Provider didn’t encouraged to call if needed Provider made patient feel alone or unattended Provider didn’t come quickly when needed
**6**	**Physical abuse**
6.1 6.2 6.3 6.4 6.5	Provider used physical force, slapped or hit the woman Woman was physically restrained Baby was separated without medical indication Didn’t receive comfort, pain relief as necessary Provider didn’t demonstrated in culturally appropriate way
**7**	**Detention in facility**

### Outcome variables

A woman was considered to have *experienced D & A* if she reported *yes* to any of the items of the seven categories. The other outcome variable was felt D & A which was labeled as *reported D & A*. This variable was based on questions at the end of all seven categories which asked women if they had felt being disrespected or abused in that particular aspect (category). If a woman responded *yes* to this question in any of the seven categories of D & A, she was considered to have *reported D & A*.

### Statistical analyses

Data was entered, cleaned and analyzed using statistical software SPSS version 17 (SPSS, Inc., Chicago, IL). Descriptive statistics were computed by reporting frequencies and percentages. Univariate and multivariate logistic regression was used to find out the determinants of reported D & A against the independent variables (place of delivery, type of provider, type of delivery, facility type as public/private, socio economic status of women and number of antenatal care visits). Odd ratios along with 95% confidence interval were given against each independent variable. Chi square was used to assess the future preferences of the respondents for location of subsequent childbirth against the reported D & A.

### Ethical considerations

The research was approved by the Institutional Review Board of AlShifa Trust Eye Hospital. Before actual data collection permission was obtained from the district administration (Health) Gujrat, and written informed consents were obtained from all the respondents ensuring autonomy and confidentiality.

## Results

### Participants’ characteristics

Total 360 women participated in the study, response rate was 100%. Their mean age was 26.4 years (SD = 4.87) ranging from 17 to 38 years. The median monthly household expenditures were PKR 15000 ranging from PKR 5000–50000. Majority of respondents had completed ten or more years of formal schooling (63.9%). A higher proportion of women were living in combined households while only 22.7% in nuclear families. Almost one in ten women was formally employed, 331 women (91.9%) were housewives. Mean number of children per woman were 2.44 (SD = 1.30) ranging from 1 to 6.

### History of antenatal care and childbirth

The dominant place of childbirth was health facilities (327, 90.8%) while 33 (9.2%) women had delivered at home. Primary gravida women were less likely to deliver at home (3, 2.9%) as compared to multigravida (30, 11.7%), X^2^(df = 1) = 5.76, p<0.05. The main provider during childbirth was a medical doctor followed by other SBA and TBAs. At home, 78.8% of deliveries were assisted by TBAs. In 11.1% cases respondents had absolutely no history of antenatal checkup. The detailed history of childbirth is given in Table 2.

**Table 2:** History of antenatal care and childbirth.

History of pregnancy and childbirth	N	%
**Delivery place for youngest child**		
Home	33	9.2
At any facility	327	90.8
Deliveries in Public facilities	110	33.6
Deliveries in Private facilities	217	66.4
**Main care provider**		
Doctor	203	56.4
Other Skilled Birth Attendant	97	26.9
Traditional Birth Attendant	60	16.7
**Type of delivery**		
Vaginal	138	38.4
Episiotomy	59	16.4
Caesarian	162	45.1
**History of antenatal (AN) visits**		
Had no AN visit	40	11.1
Had AN visit(s)	320	88.9
Less than four visits	228	71.3
At least four visits	92	28.7
**Time to reach/call provider for delivery**		
Day (8am-7:59pm)	228	63.3
Night (8pm-7.59am)	132	36.7
**Number of providers during childbirth**		
One	52	15.9
Two	103	31.5
Three or more	172	52.6
**Mother’s Parity**		
First birth	103	28.6
Subsequent birth	257	71.4

Women had gone to 55 different health facilities ranging from clinics of TBAs to secondary care hospitals in public and private sectors for childbirth. A maximum of 34 women (10.4%) have shared a common health facility for childbirth.

### Quality of amenities at health facilities

Availability of amenities at health facilities was reported to be poor (Table 3). A significant proportion of women (32.4%), who did not know where to go in the facility, reported that staff did not guide them properly about where to go inside the facility.

**Table 3:** Availability of amenities at health facilities.

Availability of amenities	N	Percentage
Bed and place to sit for women	291	89
Place to sit for attendant(s)	145	44.3
Waiting rooms	265	81
Separate labor room	281	85.9
Clean drinking water in facility	219	67.4
Facility for washing hands	201	61.5
Toilets	205	62.7
Overall clean environment of facility	197	60.2

These percentages are calculated at a N = 327

### Disrespect and abuse

Almost all women (359, 99.7%) experienced at least one type of D & A during childbirth, as determined objectively by the researchers. However, only 27.2% [95% C.I. 22.8–32.0] reported that they had felt D&A. The most reported (felt) D & A was non dignified care (44, 12.2%) while the most experienced D & A was non-consented care (Table 4). In 61.9% of cases one or more of the staff members or providers had demanded extra money as token of thanks/happiness from the woman or her family members.

**Table 4:** Experienced and reported D & A.

CATEGORY	Experienced D & A		Reported D&A	
	N	%	N	%
Non consented care	351	97.5	40	11.1
Non confidential care	211	58.6	15	4.2
Non dignified care	164	45.6	44	12.2
Discriminatory care	85	23.6	19	5.3
Abandonment of care	261	72.5	40	11.1
Physical abuse	67	18.6	2	0.6
Detention in facility	0	0	0	0
**Overall**	**359**	**99.7**	**98**	**27.2**

N = 360

### Determinants of reported disrespect and abuse

Women who delivered their child at home were significantly less likely to report D&A (n = 1, 3%) than those who had delivered at facilities (n = 97, 29.7%). Other important determinants of reporting D&A were deliveries at public health facilities, belonging to lower socioeconomic strata, less than recommended ANC visits and not utilizing same facility for antenatal checkup and childbirth (Table 5).

**Table 5:** Determinants of reported D & A- results from univariate analysis.

Determinants		Reported D & A^*^		OR	95% C.I.
		N	%		
Delivery place	Home	1	3	1	
	Facility	97	29.7	**13.49**	1.81–100.16
Delivery place	Home	1	3	1	
	Public facilities	45	40.9	**22.15**	2.92–168.06
	Private facilities	52	24.0	**10.08**	1.34–75.61
Facility type	Private	52	24.0	1	
	Public	45	40.9	**2.19**	1.34–3.59
Provider	Doctor	52	25.6	1	
	Other SBA	31	32.0	1.36	0.80–2.31
	TBA	15	25.0	0.98	0.49–1.88
Delivery type	Caesarian	47	29.0	1	
	Episiotomies	11	18.6	0.56	0.26–1.17
	Vaginal	40	29.0	0.99	0.60–1.16
Number Of AN Visits	Meeting WHO criteria	17	18.5	1	
	Not meeting WHO criteria	81	30.2	**1.91**	1.06–3.43
Time of delivery	Day	56	24.6	1	
	Night	42	31.8	1.43	0.89–2.30
Monthly expenditure	More than median	24	14.7	1	
	Less or equal to median	74	37.6	**3.48**	2.07–5.86
Household type	Combine	66	23.9	1	
	Neutral	31	38.3	**1.97**	1.16–3.34
Education status	Higher secondary or more	37	24.7	1	
	Secondary education	37	26.4	1.09	0.65–1.86
	Illiterate or primary	24	34.3	1.59	0.86–2.95
Cast of the respondents	Grade 1	40	22.1	1	
	Grade 2	36	29.0	1.44	0.85–2.43
	Grade 3 (lowest)	22	40.0	**2.35**	1.23–4.47

* Row percentages are reported, calculated by cross tabulation of predictors and Reported D&A (yes/no)

The multiple regression model showed only two significant determinants of reported D&A: place of delivery and belonging to lower income group (Table 6).

**Table 6:** Determinants of reported D & A- results from multiple regression analysis.

Determinants		OR	95% C.I.
Delivery place	Home	1	
	Public facilities	**23.54**	3.04–182.12
	Private facilities	**15.91**	2.07–122.149
Number Of AN Visits	Meeting WHO criteria (≥ 4)	1	
	Not meeting WHO criteria (<4)	1.72	0.90–3.28
Monthly expenditure	More than median	1	
	Less Or Equal To Median	**2.89**	1.63–5.11
Household type	Combine	1	
	Neutral	1.42	0.79–2.53
Cast of the respondents	Grade 1	1	
	Grade 2	1.30	0.74–2.28
	Grade 3 (lowest)	1.86	0.91–3.78

### Impact of D & A on future preferences of the respondents for location of subsequent childbirth

Women who reported D & A were four times more likely to opt for another place of birth next time for childbirth as compared to those who hadn’t reported D & A (OR = 4.37, 95% CI = 2.41–7.90).

## Discussion

The overall prevalence of reported D & A during childbirth in the study population of 360 women was 27.2%, where actually almost all women (359, 99.7%) had objectively experienced at least one of its forms. This is the highest prevalence for both experienced and reported D & A during childbirth as compared to other countries where it ranged from 70% to 98% and 16.2% to 22% respectively [10, 17]. Our findings are consistent with a study in Ethiopia where more women had actually experienced D & A (78%) but only 22% subjectively experienced it [13]. Such underreporting and/or under-recognition of D & A is common in societies where abusive behaviors and domestic violence against women are widely prevalent and acceptable [9]. Another possible explanation for the difference between experienced and reported D & A can be the denial of disrespect to protect self-esteem. Freud in its theory of Defense Mechanism explains that the normal population when exposed to anxiety producing thoughts, protects their ego and self-esteem unconsciously by denial mechanism [18].

The high prevalence of D & A during childbirth is not only against the charter of respectful maternity care but also a clear violation of basic human rights to respect and dignity. It is unethical and unacceptable in any circumstances. Moreover, it can affect maternal health adversely by discouraging women to utilize health facilities [11]. In our study women who reported being disrespected and abused during childbirth were 4 times more likely not to opt for same services again as compared to those who had not reported D & A. The findings were also validated within the study as a lesser fraction of nulliparous women had given birth at home (3%) as compared to 11.7% of multiparous women. Same discrepancy in utilization of health facilities for childbirth was reported in a national survey as well in Pakistan [6]. Almost half of the multiparous women in our sample who gave birth at home had given birth at health facilities during last their previous pregnancy. Highly prevalent D &A during childbirth can be a possible explanation of low level of facility utilization in the country for childbirth as well as for postnatal care resulting into neonatal mortality. Such statistics are alarming for a country with poor maternal and newborn health indicators.

The main determinant of reported D & A during childbirth was found to be the facility based childbirth in this study. Women who delivered at health facilities were thirteen times more likely to report D&A as compared to women who had given birth at homes. This might explain poor utilization of health facilities for childbirth in Pakistan. Shiferaw and coworkers in Ethopia explained that the reason women prefer birth at home is the more support and the trust they get from the TBAs [19]. In our study women whose deliveries were assisted by TBAs had reported lesser D&A although statistically not significant. Women even when receiving technically sound care but lacking in emotional support perceive it as low quality care [20]. Reported D & A in public hospitals was twice as high compared to private hospitals (OR = 2.197, CI = 1.34–3.59). Unpleasant experiences like ignoring or the delayed response and lack of communication in public sector make patients agonized rather than treated [21]. This high prevalence of D & A in public facilities is a possible explanation that public facilities are utilized for only 15% of total childbirths in Pakistan [4].

Socioeconomic disparities were observed in vulnerability to D & A during childbirth in our study. Women with low socioeconomic status were almost three times more likely to feel disrespected and abused (OR: 2.89, C.I. 1.63–5.11). Similar pattern is reported in Kenya where poorest wealth quintile had reported more D & A as compared to richest quintile [22]. Previous evidence from Pakistan shows that more than 60% of maternal deaths occur in low income groups [23]. Univariate analysis also showed that women belonging to lower caste were more disrespected and abused as compared to higher caste women. D & A can adversely affect the maternal and newborn health in this population group which is already vulnerable to poor health owing to poverty and social exclusion. There is need to focus on social determinants of health and to train healthcare workers to provide respectful and equitable care in the country.

It is reported that almost half of childbirths happen at home in Pakistan [6], but in this research only 9.2% deliveries were home based. Another study conducted in the same district also reported similar findings as most of women in Gujrat tend to deliver at hospitals (81.2%) rather than at home [24]. The reason behind this contrast is that Gujrat is among the ten most developed districts of Pakistan. The health indicators of Gujrat are also better when comparing the sum indicator of whole country, MMR = 172 as compare to MMR = 276 [16]. This indicates relatively better provision of health services and a trend to deliver at facilities than home.

The most commonly experienced D & A was non consented care and lack of informed choice (99.7%) followed by abandonment of care (72.5%) and non-confidential care (58.6%). The evidence clearly follows the study of Asefa and Bekele (2015) where a lack of information and informed choices (94.8%) and abandonment of care with 39.3% prevalence dominated other categories of D & A [13]. The second most experienced and reported category of D & A was the abandonment of care with 72.5% and 11.1% respectively. This is clearly against the guidelines to the respectful maternity care that states that any woman who is in labor or just delivered a baby should never be left alone or without care at all [25]. The most commonly felt D & A was the non-dignified care where women had experience verbal insults, abusive language and rude attitudes. This is unethical and a clear violation of basic human rights. Such rude behaviors, negligence and carelessness on the part of the providers may not just have negative maternal outcomes but also the long lasting psychological impacts. Women remember the process of childbirth and the events of the surroundings for many years [26]. It means that lack of respectful care can build an everlasting hesitation and reluctance for future utilization of health services. Non responsive attitude of the providers ultimately results in a situation where health care facilities are only utilized for complications [27].

### Limitations

This study is based on the data from women residing in rural setting, statistics may be a bit different in urban settings. However, we expect a small variation as women in our sample had consulted health facilities of both urban and rural areas. The main limitation of the study was a relatively lesser portion of women who had given birth at homes and even a small fraction of them reported D & A, which resulted in smaller cell sizes and wider confidence intervals in statistical analysis. Similar studies in settings where proportion of home based deliveries is higher will delineate the true impact of facility based D & A.

## Conclusion

Disrespect and abuse during childbirth is highly prevalent in Pakistan, although remains under recognized and under-reported. The most commonly reported D & A was violation of women’s right to respect and dignity, followed by lack of informed choices and consent, and the abandonment of care. More than half of the women reported financial abuse as well. Main determinants of D & A were facility based childbirth and low socioeconomic status of women. Women felt more respected and cared for if they had given birth at homes as compared to health facilities. The risk of being disrespected and abused was twice in public health facilities as compared to private. D & A during childbirth was found to be a strong factor contributing to choice of facility/provider for next childbirth. We suggest that facility based D & A during childbirth might be the missing link in understanding the low level of SBA in Pakistan. Targeted interventions are needed to deal with the highly prevalent issue of D & A to improve maternal health in the country. Maternal health policies in Pakistan need to be revised based on the charter of respectful maternity care.

## Supporting information

S1 Dataset SPSS file for anonymised data. (SAV)
